# Former suicidal inpatients’ experiences of treatment and care in psychiatric wards in Norway

**DOI:** 10.1080/17482631.2018.1461514

**Published:** 2018-04-13

**Authors:** Julia Hagen, Birthe Loa Knizek, Heidi Hjelmeland

**Affiliations:** a Department of Mental Health, Norwegian University of Science and Technology (NTNU), Trondheim, Norway; b The Regional Center on Violence, Traumatic Stress and Suicide Prevention (RVTS), Region Mid-Norway, St. Olavs Hospital, Trondheim, Norway

**Keywords:** Care, psychiatry, qualitative research, suicide, treatment

## Abstract

**Purpose:** The purpose of this study is to explore how former suicidal inpatients experienced treatment and care in psychiatric wards in Norway following the implementation of the *National guidelines for prevention of suicide in mental health care*. The focus of the analysis was on aspects of treatment and care with potential for improvement. **Method:** We interviewed five former inpatients and analysed the data by means of Interpretative Phenomenological Analysis. **Results:** Experiencing a sense of companionship with the staff and receiving individualized treatment and care was important for the participants. This involved establishing trusting connections with mental health workers who treated them with respect, made them feel valued, and who recognized their suffering and needs. The formerly suicidal patients experienced being in a recovery process, which was promoted by the support of mental health workers. Although the participants reported mostly positive experiences, there were examples of insufficient care. Sometimes, they felt that their suffering and suicidality were not sufficiently recognized. **Conclusion:** Our study indicates that although there has been increased focus on suicidality in the mental health services, among other through clinical guidelines, some mental health workers still lack competence and should focus more fully on how to provide individualized care for suicidal inpatients.

## Introduction

Many of those who are admitted to psychiatric hospitals struggle with suicidality. For instance, a Norwegian study found that about 52% of first-time admissions to psychiatric hospitals were related to suicidality (Øiesvold, Bakkejord, Hansen, Nivison, & Sørgaard, 2012). Mental health workers face the task of helping these patients through their suicidal crisis and preventing suicide attempts and suicide. However, preventing suicidal acts is challenging, and in 2015, about 27% of the suicides in Norway occurred among patients or recently discharged patients of specialized health services (Saastad, 2016).

The *National guidelines for prevention of suicide in mental health care* were published in 2008 with the purpose of making mental health workers more competent and confident in dealing with suicidality among patients (Norwegian Directorate of Health and Social Affairs, 2008). Although the guidelines have raised mental health workers’ awareness about suicidality, the main emphasis is on the assessment and management of suicide risk and not on how to provide good care to suicidal patients. Previous research has shown that patients appreciate being cared for by empathic health workers who value them as individuals and respond to their needs (Berg, Rørtveit, & Aase, 2017; Lees, Procter, & Fassett, 2014; Sun, Long, Boore, & Tsao, 2005; Talseth, Jacobsson, & Norberg, 2001; Talseth, Lindseth, Jacobsson, & Norberg, 1999; Vatne & Nåden, 2014) and who inspire hope and move them from a ‘death-oriented’ position to a ‘life-oriented’ position (Cutcliffe & Stevenson, 2007; Cutcliffe, Stevenson, Jackson, & Smith, 2006). Berg et al.’s (2017) review shows that, for suicidal inpatients, safety means ‘feeling safe’, which involves establishing close connections with the professionals, being protected by supportive staff and re-establishing a sense of control over their lives. Some patients have reported that experiences of not feeling valued have contributed to increased suicidal behavior while they were hospitalized (Samuelsson, Wiklander, Åsberg, & Saveman, 2000; Talseth et al., 1999). Unmet needs for connection, protection and control can make the patients feel unsafe and increase their suicidality (Berg et al., 2017). Good care implies more than identifying and managing mental disorders and suicide risk, which has been established by several clinicians and researchers in the field (Cutcliffe & Santos, 2012; Jobes, 2006; Michel, Valach, & Gysin-Maillart, 2017; Rogers & Soyka, 2004). Considering the increasing focus on suicidality in mental health services over the last decade, there is a need to investigate the experiences of people who have been admitted to psychiatric hospitals because of suicidality.

The purpose of this study is to investigate how persons admitted to psychiatric hospitals because of suicidality experience treatment and care in mental health wards since the implementation of the *National guidelines for prevention of suicide in mental health care* about 10 years ago (Norwegian Directorate of Health and Social Affairs, 2008). More knowledge from the service-user perspective may contribute to increasing mental health workers’ understanding of how their treatment and care are experienced by suicidal patients and thus improve suicide prevention work in the psychiatric wards. Our research questions are: How did (former) suicidal inpatients experience treatment and care in psychiatric wards following the implementation of the *National guidelines for prevention of suicide in mental health care*? What can be improved in the treatment and care of suicidal inpatients?

## Method

This is a qualitative study. We collected data through individual and semi-structured interviews and analysed the material by the means of Interpretative Phenomenological Analysis (IPA) (Smith, Flowers, & Larkin, 2009; Smith & Osborn, 2003).

### Participants

The participants were five persons (four females, one male; aged 33–54 years) previously hospitalized in psychiatric wards because of suicidality. Two had been admitted to a psychiatric hospital for the first time and were hospitalized for about 3 months. Three had been admitted several times over the past 15 years. Their most recent hospitalizations lasted for about 2–3 weeks. All were admitted to an acute ward before being transferred to a district psychiatric center. At the time of the interviews, it had been from 1 week to 9 months since the participants’ last hospitalization. Three were admitted because of a suicide attempt (self-poisoning or hanging), and two were admitted because they were close to attempting suicide.

### Recruitment procedure

Some nurses in selected acute psychiatric wards and therapists in selected psychiatric outpatient units in mid-Norway assisted in recruiting the participants. The first author provided the staff with an information letter about the study describing the inclusion criteria: former patients over 18 years old who had been admitted to an adult psychiatric ward because of suicide attempt or severe suicidal thoughts during the last 12 months. People who had one or more admissions and people who had been hospitalized repeatedly because of recurrent suicidality could also participate in the study. The research ethics committee approving the study determined the recruitment procedure. Therapists working in out-patient services informed potential participants about the study and asked patients whether the first author could contact them by phone to ask if they wanted to participate. Two patients consented to be contacted by the first author and agreed to participate. Additionally, selected staff members working in acute inpatient units identified potential participants, informed them about the study, and gave them an information letter. Those willing to participate had to contact the first author after they had been discharged. Three of the participants made contact by phone and agreed to participate. The strategy for purposefully selecting participants was influenced by homogenous and criterion sampling (based on their psychiatric hospitalization because of a suicidal crisis within the last 12 months) and convenience sampling (based on accessibility) (Patton, 1990).

### Interview procedure

The first author conducted the in-depth, semi-structured interviews of the participants, who could suggest or decide on the time and place for the interview. She interviewed three participants in an office/meeting room at her workplace, one participant in a meeting room at a psychiatric outpatient service, and one participant in the person’s home. Four interviews lasted from 85 to 114 min, whereas one interview lasted 31 min. All interviews were recorded and transcribed verbatim. The first author used an interview schedule as a tool to help guide the conversation (if necessary) to obtain relevant information about the participants’ experiences of suicidality and being hospitalized in a psychiatric ward. The main questions in the guide were: Can you please tell me about your situation and how you felt when you were admitted to hospital? How did you experience being hospitalized after attempting/contemplating suicide? How did you experience your encounters with the professionals (mental health nurses and therapists)? What does a good connection/relationship with a professional mean to you? Can you please describe a situation where you did/did not experience a good contact/relationship with a professional? What does good care mean to you? What do you think was most important to you during the hospitalization? In keeping with Smith et al. (2009), the interviewer probed for further description (e.g., Can you please tell me more about it? How did you feel?) to go deeper into the participants’ experiences and to obtain more specific and detailed descriptions. The interviewer used confirmatory/interpreting questions (e.g., So you felt/thought… ?) in an attempt to clarify the participants’ experiences and views (Kvale & Brinkmann, 2009). At the end of the interview, she asked the participants how they had experienced the interview.

### Analysis

We analysed the interview data by the means of Interpretative Phenomenological Analysis (IPA) (Smith et al., 2009; Smith & Osborn, 2003). The approach is suitable for in-depth analysis of data from a small sample and is commonly used to interpret experiences of illness or psychological distress, particularly experiences that are of existential importance to the participant (Smith, 2011). IPA’s theoretical underpinnings are phenomenology, hermeneutics and idiography, which calls for a focus on a close interpretative engagement of particular instances of lived experience and the meaning the participant makes of those experiences (Smith et al., 2009). The first author conducted all steps of the analysis. To increase the trustworthiness the second and third authors were involved in the process of interpreting data. In keeping with IPA (Smith et al., 2009) the approach involved the following (simplified) steps: 1) All authors read and re-read the transcripts to become familiar with the participant’s personal experiences of suicidality and receiving mental health care. 2) The first author created a document with relevant meaning units from each transcript according to the focus of the study. 3) Since all the participants’ narratives contain descriptions of their life before hospital admission, during and after the hospital stay, the first author organized the content according to the timelines a) before hospitalization, b) during hospitalization, and c) after discharge/life now. Then, she developed a number of preliminary themes (e.g., existential struggle, suicidal crisis, feeling worthless, relational problems, good ‘chemistry’ or not with staff members, being seen and taken care of, not being understood or recognized, resources, taking responsibility, seeking meaning in life, ongoing processes of pain and recovery), which was discussed with the second and third author. 4) We continued to immerse ourselves in the material, and the first author developed three overarching themes (the suicidal crisis is building up, experiences of support/lack of support from professionals, suicidality and recovery processes of pain and self-development), and searched for connections across the themes. In keeping with the research questions, we then focused on the participants’ experiences of support/lack of support from professionals and their recovery process. Based on these two overarching themes and the transcripts, we developed the final descriptions related to the connection between the suicidal patient and mental health workers (Theme 1—*Seeking a sense of companionship to feel safe to share their suffering and suicidality*), and related to the participants’ experiences of treatment and care (Theme 2—*Seeking individualized treatment and care to feel recognized as a valuable person*), and related to the participants’ personal recovery process (Theme 3—*Seeking support to promote their recovery process*). The participants’ experiences of suicidality were linked to their experiences of treatment and care in psychiatric wards, and their recovery process. To contextualize our findings, we also describe some characteristics of their suicidal crisis as they experienced it. 5) The first author had worked with each narrative in depth before moving on to the next transcript. Subsequently, all authors collaborated in looking for patterns, similarities and differences across the data to develop more general descriptions. We thoroughly discussed the material, the interpretations of the data and several drafts of the findings during several meetings until all authors agreed upon the final three themes and descriptions. A simplified example of the analytic approach is illustrated in Frame 1.

**Frame 1.** An extract from one interview with preliminary notes and themes, and the development into to one of the overarching themes and final descriptions.

Original transcript	
**Interviewer**: *Mmm, but can you tell me a bit more about that, because*	
*you say that having conversations, you want them to be helpful. But did*	
*you sometimes experience that it helped and sometimes that it didn’t or..?*	**Preliminary notes and themes**
**Susan**: Yes, sometimes it helped and sometimes it did not. And that has a lot, eh that has a lot to do with personal chemistry, for instance. There were someone who in a way said “yes but, yes that’s how it is having depression”, but that isn’t always helpful. And sometimes it helped that they just listened to what I had to say. And other times they put me on a different track. It seems as if many had good strategies to handle me and others had not. I had a pretty, just before I was discharged I had a terrible day where I could not speak to him the contact [among staff] I had that evening. So I ended up harming myself, cutting my thighs. And then I went to talk to the night staff, because I thought that I have to hold on to the night shift is coming and then I can talk to her. And she said “well, well”, I said I had a very bad night and that I was worried in a way before I was going to bed. And then she said “yes, it goes up and down for all of us in life, you know”. And then I tried to communicate that “I am really having a hard time now”, and then she said, “yes, but you have to think like [a Norwegian singer-songwriter], be an optimist”. And I interpreted it more like yes, “pull yourself together”. And then I just finished politely and smiled and said, “thanks for the conversation” and went to bed, that is, I went and sat down in the living room after I had taken medicine until I became so tired that I was sure to get to sleep when I went. So it was a little up and down how the conversations helped. However I mostly felt that it helped to have conversations.	Helpful conversations has a lot to with personal chemistry and connection with the professional. Focusing on mental disorder (depression) is not helpful. To be listened to is helpful. Thinks staff has different strategies to handle patients. Lack of connection with the contact among staff led to withdrawal and self-harm. Lack of understanding from the nurse. The suffering was not recognized or it was dismissed. Was left to take care of herself. Has both positive and negative experiences of the conversations with the staff.
**One overarching theme: Experiences of support/lack of support from professionals** This and other interview excerpts show that the participants experienced both support and lack of support from the professionals. Although they reported many positive experiences of being taken seriously, being listened to and taken well care of, they had examples (as illustrated in the excerpt above) of that their suffering and suicidality were not sufficiently recognized or responded to in a helpful way. Sometimes, it appeared that the staff focused much on mental disorders (e.g., depression) and/or they distanced themselves from the patients’ difficulties and suicidality, and it seemed as if they did not spend enough time to explore and respond to each person’s particular needs.	
**One final description: Seeking individualized treatment and care to feel recognized as a valuable person**	
Dwelling into the transcripts and reflecting further on the participants’ experiences led to the development of the final descriptions “seeking individualized treatment and care to feel recognized as a valuable person”, which entails that suicidal inpatients need to encounter professionals who treat them with respect, who are sensitive and recognize their suicidality and needs, and who make them feel like valuable fellow human beings.	

In keeping with Smith et al. (2009), we attempted to ‘bracket’ prior knowledge and assumptions, particularly in the initial analysis, in an effort to be more attentive to the participants’ unique experiences and to prevent one’s own prior knowledge from blocking new insight. In this reflexive and hermeneutical process—moving between data, our understandings, and descriptions and the literature—we have attempted to stay close to the participants’ narratives.

### Ethical considerations

The Regional Committee for Medical and Health Research Ethics approved the study. The participants signed an informed consent form. They were informed that they could withdraw from the study at any time prior to publication without giving a reason. The participants had the opportunity to contact the researcher after the interview if they had any questions about the study or their participation or if the interview had evoked mental distress and they needed a follow-up consultation. The first author had arranged for a mental health nurse at a psychiatric liaison service at the general hospital to be available for consultation at short notice. None of the participants needed follow-up after the interview. We treated the data in a confidential manner and present the information about the former inpatients in such a way that they are not identifiable. We refer to all the participants with fictitious female names or as ‘she’ to protect their anonymity, including the one male participant.

## Findings

The participants reported more positive than negative experiences with the treatment and care in the psychiatric wards. Our main focus here is on aspects that could be improved based on what the participants found important. The findings consist of three themes: ‘seeking a sense of companionship to feel safe to share their suffering and suicidality’, ‘seeking individualized treatment and care to feel recognized as a valuable person’, and ‘seeking support to promote their recovery process’. To contextualize our findings, we describe some aspects of the participants’ suffering and suicidality and give voice to some of their pain before we move on to describe the themes. Although the participants’ suicidality evolved from very different life situations, their narratives reflect self-devaluation and loss of self-worth. The participants shared thoughts of being ‘monstrous’ and of no worth and described patterns of devaluing their achievements, undervaluing their role as a spouse or parent, or having feelings of being a burden or replaceable. For instance, Katie said: “I start to pull myself down […] And think that, no, I am not worth anything. I look at myself as a freak, and I’m not going to be OK. I will never be OK”. It is as if she felt that she did not deserve to get better, that she is not worthy a better life. In different ways, the participants struggled with their basic sense of self-worth. The participants shared experiences of disconnecting from people and from life itself, as explained by Ellen: “The desire to live, it disappears completely. In the end I feel that it is better for my kids, it is better for my husband, it is better for my in-laws that I am dead”. Being suicidal was like being in one’s own world or “bubble”. Their life world was constricted, and even close family members were disregarded. The following is a description of findings with excerpts from the interviews.

### Seeking a sense of companionship to feel safe to share their suffering and suicidality

The participants emphasized the importance of having a good connection with the care provider, and they noted that ‘good chemistry’ was an important part of such a connection. Ellen described: “it is not everyone you talk to about everything then. It has a little bit to do with chemistry as well. They you feel you get the best contact with”. The participants pointed to not being able to talk confidentially with just anyone. It seemed as if ‘good chemistry’ or a good connection involved a sense of companionship with the mental health worker, which involves closeness and trust. Such a connection or contact entails feeling safe and comfortable enough to open up and talk about personal issues including suicidality, as expressed by Liz: “it feels very safe to have NN [the therapist], because he has … knows me and my whole situation […] Yes, so that is very safe then. So, I feel like I can say anything to him then”. She had established a relationship with the therapist several years ago and appreciated that he was her regular therapist when she was hospitalized. The connection with the therapist represented safety for her, and she could talk about anything with him.

Sometimes, the connection or the ‘chemistry’ with the mental health worker did not feel right. For instance, there was a sense of a lack of trust, which meant that the participant did not feel safe or did not want to approach the person for support in times of distress. Susan revealed trying to strangle herself in her room at the hospital and not wanting to talk to the member of the staff assigned to her that day: “yes, I would not tell it to my contact because I did not have good chemistry with him”. The mental health worker did not represent the safety and care she needed, and she therefore chose not to share her pain and suicidal experiences with that person. Liz and Susan admitted to trying to change one of their contacts among the nursing staff with whom they did not feel a good connection, but their request was denied. Liz explained: “because I think it is important that those who are in one’s own treatment group are someone one has good chemistry with. So, because when they are on duty, they are assigned to you, but then, but they would not [change her contact]”. She pointed to the importance of connecting to the staff in her treatment group. However, even if the patients had tried to change a staff member assigned to them, they had to relate to him/her after the request was denied, which was challenging.

This theme shows that for the suicidal patients in this study, a good connection with the mental health workers entails a sense of companionship that enables them to approach the professionals and share their suffering and suicidality when needed. If there is a lack of companionship with the mental health workers and lack of trust, the suicidal patients may withdraw and keep their pain and suicidality to themselves, which might be harmful for the patients’ mental health and safety.

### Seeking individualized treatment and care to feel recognized as a valuable person

Individualized treatment and care involved encountering mental health workers who took them seriously and who treated them with respect, who was sensitive and recognized their suicidality and needs, and who made them feel like valuable fellow human beings. Individualized treatment and care also entailed a good connection with the mental health worker, i.e., a sense of companionship with the professional. Thus, this theme relates to the former theme described above. Susan shared the following example indicating an individualized approach:

“She [the nurse] just came up to me and, ‘Yes, I see you are tired now, and it’s all right. Just be tired’, and I thought that was so good. And it was she who found me with [the means to attempt suicide] that night. [She] sat down and instead of in a way, it was someone I felt in a way … accused me a bit sometimes, not accused but sort of like, ‘it is foolishness to engage in such things’, while she was a little more like, ’yes I understand you are in pain, or I can’t really understand how you are doing, but it will get better, I am sure you can make it’. And at the same time somehow, yeah, just was a comforting fellow human being”.

Her suffering was recognized and she felt comforted as opposed to other times where she felt she was being treated in a more judgmental way. She valued feeling some kind of companionship with the care provider who offered more of herself than professional expertise. This was also experienced by other participants, as described by Ellen: “he [the therapist] saw me, as I was […] in a way, he is somewhat like a fellow human being and not just a doctor or a therapist. But he, yes, gives a little bit more of himself”. In order to help the patients feel valuable and unique, it seemed important that the mental health workers used their personal qualities and acted as fellow human beings rather than only professionals.

Katie thought the staff focused more on suicidality and were more supportive now than they had been 15 years ago. This may be related to the increased focus on suicide prevention following the implementation of the *National guidelines for prevention of suicide in mental health care* (Norwegian Directorate of Health and Social Affairs, 2008). However, although Katie was satisfied with this development, she also noted that the staff should go even deeper into the patient’s situation: “and they [the mental health workers] have started to go deeper into it. I think they are somewhat deeper now, […] But perhaps even more. […] Go deeper in on the patient. Yes, a bit like … Take the patient seriously. Go deeper into the situation, the thoughts”. The mental health workers appear to have increased their knowledge and understanding, but it seems they can become even better at exploring the complexity and depth of the person’s suicidality. The participants reported several experiences of not being taken seriously. Susan described:

“… I said I had a very bad night and that I was worried in a way before I was going to bed. And then [the nurse] said ‘yes, it goes up and down for all of us in life, you know’. And then I tried to communicate that ‘I am really having a hard time now’, and then she said, ‘yes, but you have to think like [a Norwegian singer-songwriter], be an optimist’. And I interpreted it more like yes, ‘pull yourself together’. And then I just finished politely and smiled and said ‘thanks for the conversation’ and went to bed, that is, I went and sat down in the living room after I had taken medicine until I became so tired that I was sure to get to sleep when I went”.

This statement shows a lack of recognition from the nurse, who did not respond according to the participant’s needs. It seems the nurse did not get the message or that she dismissed the patient’s need for help through quick (albeit not constructive) advice. Susan was left to deal with her pain alone, mainly through the use of sedatives. She revealed that she made several suicide attempts during the hospitalization, and an important question is whether some of the attempts could have been prevented if her suffering and suicidality had been sufficiently recognized by the care providers. The participants expressed that they at times wanted to talk more about their suicidality and the background of their problems, but they experienced that staff did not have enough time to work thoroughly with their problems. Moreover, some staff members seemed reluctant to talk about suicidality. Susan experienced: “they also told me that ‘it [suicidal thoughts] is something we do not want to talk about too often because it may give you ideas. If you are not there, we shall not induce those thoughts in you’, but perhaps it would have helped me to try to talk about it sometimes”. Thus, it appears that in spite of the efforts to increase the knowledge of suicidality in the services (e.g., through clinical guidelines), some mental health workers still believe in the myth that talking about suicide could evoke suicide thoughts and increase the risk of suicide.

Others reported experiences of insufficient care related to being met with an emphasis on mental disorders and treatment with medication. The participants had been assigned one or several psychiatric diagnoses, but they seemed to doubt whether the diagnoses were correct. Ruth found it difficult to accept the psychiatric diagnosis assigned to her:

“I really got very good information […] about depression, about the first step of taking medicines was also a big step for me. And maybe accepting that I was ill. Taking the first pill was enormously difficult. […] Because it really affected me to accept that. And reading the brochure where it is explained very much as a disease. I did not feel ill. I just felt sad, that I did not want to live anymore. That is something completely different from being ill in the head. So, accepting that is terribly difficult”.

Ruth perceived that the mental health workers understood and approached her suicidality as part of a presumed underlying disorder, which she did not acknowledge. Rather, she felt that her own perspectives of the suffering and suicidality were not taken as much into account as they should have been. Furthermore, the participants questioned the usefulness of the medication they received and said they lacked follow-up consultations about their treatment from the physician/psychiatrist. Both Susan and Ruth said they experienced a deterioration in their mental state when they started taking antidepressants (e.g., increased impulsivity with regard to suicidal acts), and several participants reported negative side-effects (e.g., blunted emotions/feeling indifferent) and/or lack of efficacy. Ellen had used various psychopharmaceuticals for many years but had stopped taking them over a year ago, and she felt that this had had a major impact on her quality of life: “I got my life back after I quit taking medicine. Very literally, I have … And they see it—my family—that I … now I live; I [only] existed before”. This is a powerful statement about the potential negative effects of psychotropic drugs. Ruth suggested that mental health workers’ belief in medicine is perhaps inflated: “the only thing I really doubt a little, that has actually to do with the medication. […] That there is really no evidence that it helps. So why do they offer that as the only solution? In addition to—or, maybe it is a bit overrated then. They could have been a little more honest about that”. She wondered why medication seemed to be the main element of the treatment plan even though the effect seems uncertain. She indicated that mental health workers are aware of the uncertain effects of medicine and wished they were more honest.

This theme illustrates suicidal patients’ need to be met by mental health workers who emphasize an individualized approach, i.e., who recognize the patients’ basic value as fellow human beings and who discern and understand their particular needs. However, in this study, the suicidal patients sometimes felt that they were not sufficiently recognized by the care providers. Additionally, they experienced a lack of efficacy or adverse effects of psychotropic drugs. The experiences are examples of insufficient treatment and care, and it seems as if the suicidal patients at times felt that their perspectives and experiences were overlooked, dismissed or overshadowed by the mental health workers’ (medical) ideology and clinical routines.

### Seeking support to promote their recovery process

The participants’ experiences illustrate that they were in a process of development and change involving existential, relational, and practical aspects of their lives. The experiences appeared to be a part of a personal recovery process that was influenced by the support they received form mental health workers, and that continued after they were discharged from psychiatric hospital. Experiencing suicidality and receiving treatment and care had increased their ability to talk about personal issues and their self-awareness, as illustrated by Susan: “I think it is one of the best things that has happened to me. I have gotten so much more insight into myself. I have become so much more open about personal things. I have started to appreciate a lot more, I think, I think I will appreciate people I have around me more after this when I somehow start coming back”. Something was gained through the suffering and the mental health care, which contributed to a development on an individual and relational level. Susan was starting to appreciate other people more, or hoped that she would when she started to “come back”, indicating that this was an ongoing process. Ruth shared similar experiences, although she seemed to struggle more to find meaning in life: “I think I need to go deeper, and find it then. Something that can give me some meaning, really. But I haven’t given up. Otherwise, I would not have been here today. Like that. But all in all, oh my, I have learned so much in this past year. Because before I was the kind of person who lived. And walked in the spiral without thinking for a second what it was all about”. The process of recovery involved reshaping the sense of self and life. In the quest for meaning, Ruth had, among other things, immersed herself in philosophical and spiritual literature, and both she and Susan had benefited from practising mindfulness.

Working on relationships with family members, particularly their spouse and children, seemed to be an important part of the recovery process. Some relationships appeared to be changing, and some relationships had to be improved or repaired. Ellen described how she, with the support of her therapist, had made an effort together with her husband to create and implement a safety plan:

“And I have, actually together with my husband, made a plan for the whole family and everything. Which is divided into three different phases. [The first] is like a healthy/normal phase; that is green. And then there is a yellow phase; then the sleeping problems occur. And some things that perhaps my family must do if I can’t manage to do things. And then there is a red phase; then I am ill. And we have inserted what do we do, how do we do things, who does things, when… […] and how do we get it back to … because we would rather be on green then”.

She had included the whole family in the safety plan, which was very helpful to her in vulnerable phases. The plan included an awareness of the warning signs of deterioration in her mental health and supporting strategies from both family members and mental health workers. Katie had experienced that the process of suicidality and hospitalization had helped her to get her own housing and an extended network in the community: “she [the psychiatric nurse in the community] has helped me so that I can go to a meeting center there. I have a tendency to isolate. […] So, I feel that they have made a proper network around me that I have not had before”. The recovery process involved making new connections with other people. This made Katie less isolated, and she appeared to be more content with life.

This theme indicates that experiencing suicidality and receiving mental health care can involve personal development and change that is part of a recovery process that continues after being discharged from psychiatric hospital. Although suicidal patients may be vulnerable, they have strength and abilities to change or develop themselves and their lives for the better. In this study, the participants had benefited from the support of family members and mental health workers, and although they reported that some aspects of care could be improved (described in previous themes), they positioned themselves as having the primary responsibility for their own recovery process.

## Discussion

The findings in this study indicate that suicidal inpatients emphasize to encounter mental health workers who make them feel safe and comfortable enough to open up and share their suffering and suicidality, and who recognize them as valuable persons and respond to their particular needs. It appears that to achieve good connections with suicidal patients so that they can feel a sense of companionship, and to provide individualized treatment and care, mental health workers need to use their personal qualities and act as empathic fellow human beings. Further, our study suggests that suicidal inpatients experience a process of recovery that can be promoted by the support of mental health workers. However, even though the former suicidal inpatients shared mostly positive experiences, there are examples of insufficient or poor treatment and care where the patient did not experience a good connection with staff members, and the suffering and suicidality were not sufficiently recognized and responded to. Our study suggests that insufficient care could lead to suicidal patients withdrawing and keeping their pain and suicidality to themselves, which might be harmful for their well-being and safety.

Our findings relate to two prior Norwegian studies about suicidal inpatients’ experiences of being cared for by nurses (Talseth et al., 1999) and physicians (Talseth et al., 2001). In Talseth et al.’s studies, experiences of good care were described as being confirmed (e.g., the patients and their needs were acknowledged and attended to), and experiences of poor/insufficient care were referred to as not being confirmed (e.g., the patients and their needs were overlooked) (Talseth et al., 2001, 1999). It appears that although the studies of Talseth and colleagues were conducted almost two decades ago, and the mental health services have undergone changes with regard to increased focus on suicide prevention through the National guidelines (Norwegian Directorate of Health and Social Affairs, 2008), our findings are quite similar to theirs (Talseth et al., 1999, 2001). As then, participants in our study shared examples of their needs being overlooked. For instance, one participant experienced that her request for care was dismissed with superficial advice (specifically, “be an optimist”). In 1994, Neimeyer & Pfeiffer made a list of common errors in the way mental health workers respond to suicidal patients, and giving advice, relying on superficial reassurance and employing passivity (avoiding actively engaging in the patient’s distress) were on that list that is now 24 years old. Caring for suicidal inpatients may be emotionally demanding (Hagen, Knizek, & Hjelmeland, 2016). Thus, providing insufficient care, such as giving superficial advice, may be a way of distancing oneself from strong negative feelings (Neimeyer & Pfeiffer, 1994) and avoiding the discomfort evoked by suicidality (Carlén & Bengtsson, 2007; Talseth, Lindseth, Jacobsson, & Norberg, 1997). However, as our study illustrates, suicidality may be experienced as a crisis of the self (Webb, 2010), involving weakened self-worth or a total loss thereof (Orbach, 1997; Shneidman, 1980), and suicidal acts may be a way of escaping from aversive self-awareness (Baumeister, 1990). It is therefore crucial that mental health workers aim at reducing the psychological and emotional pain and work to strengthen or recognize the suicidal individual’s self-worth. Our findings are consistent with Berg et al. (2017) who found that patients’ connections with the professionals made them feel valued and acknowledged as human beings. Previous studies have pointed to that recognizing the suicidal patient—in terms of ‘you exist, you are not alone and your experiences are acknowledged’—is essential (Cutcliffe et al., 2006; Talseth et al., 1999). Such care may increase the person’s belief that he/she matters to others in the world, which has been associated with increased self-esteem and decreased suicide ideation (Elliott, Colangelo, & Gelles, 2005). Our findings add to this literature by indicating that in order to provide good treatment and care and to make the patient feel valuable, it is important that the mental health workers employ their personal qualities as empathic fellow human beings and provide the patients with a sense of companionship. Dierckx de Casterlé (2015) used the concept ‘skilled companionship’ to describe a kind of care which involves that health workers are emotionally and existentially close to the patient, and where the relationship between them is characterized by mutual trust and equality.

Our findings show that in spite of vulnerability and suicidality, the participants communicated strengths and resources to change their lives for the better. The participants had learned much through their suffering and were still in a process of recovery. Mental health workers can promote the process of finding meaning and learning from the suffering (including suicidality) (Cutcliffe, Hummelvoll, Granerud, & Eriksson, 2015). The suicidal patients’ resources and continuing recovery process is more evident in the present study compared to what is described in several other studies (e.g., Berg et al., 2017; Talseth et al., 1999, 2001). However, Sellin, Asp, Wallsten, and Wiklund Gustin (2017) have explored how persons at risk of suicide experience the process of recovery. Their findings show that recovery means ‘reconnecting with oneself while struggling between life and death’ (Sellin et al., 2017). Many years ago, Carl Rogers wrote about ‘client-centered therapy’, later transforming it into the ‘person-centered approach’ (Rogers, 1980). He pointed out that his clients seemed to struggle with the questions: ‘Who am I, really? How can I get in touch with this real self, underlying all my surface behavior? How can I become myself?’ (Rogers, 1961, p. 108). These questions appear very relevant for suicidal persons, including the participants in our study who seemed to struggle with such existential issues. Sellin and colleagues (2017), pointed out that recovery from a suicidal crisis involved reconnecting with oneself and questioning how life could become worth living. Further, this process is facilitated in mutual connections with professionals and supportive relatives (Sellin et al., 2017). Rogers’ key message was that clinicians could promote a person’s change and growth by being empathic listeners, understanding, genuine, and accepting (Rogers, 1980). This philosophy is found among other researchers and clinicians who have argued for a person-centered approach with an emphasis on the patient’s perspectives, a trusting mental health worker-patient relationship (Cutcliffe & Stevenson, 2007; Jobes, 2006; Leenaars, 2004; Rogers & Soyka, 2004), and a narrative approach wherein the person can talk and reflect about their suicidality (Michel & Valach, 2011; Michel et al., 2017). However, in times of managed care and busy schedules, such aspects may be neglected or not prioritized (Michel, 2011). Rather, mental health workers may rely on questionnaires and manualized interviews (Michel et al., 2017) and spend too much time on diagnostics and other clinical procedures as we found in a previous study (Hagen, Hjelmeland, & Knizek, 2017).

In our present study, some participants expressed doubts about the psychiatric diagnosis/diagnoses assigned to them, and four of them reported a limited or ineffective response to antidepressants and other psychopharmaceuticals in addition to negative side-effects. In a recent systematic review, Jakobsen et al. (2017) concluded that the potentially limited beneficial effects of antidepressants (SSRIs) appear to be outweighed by harmful effects. Critical voices have been raised against diagnostic practices and the potential adverse effects of psychiatric medication (Gøtzsche, 2013; Whitaker, 2010), and several authors have questioned the overreliance on biomedical models with regard to how we understand and manage suicidality (Cutcliffe & Santos, 2012; Hjelmeland, Dieserud, Dyregrov, Knizek, & Rasmussen, 2014; Marsh, 2016; Webb, 2010). Even the assumption that 90% of all suicides are associated with mental disorders (Cavanagh, Carson, Sharpe, & Lawrie, 2003) is strongly challenged by researchers who argue that there is no valid evidence for this 90% statistic (Hjelmeland, Dieserud, Dyregrov, Knizek, & Leenaars, 2012). However, psychiatry continues to be heavily influenced by a biomedical ideology that in turn affects how mental health workers understand suicidality and approach suicidal patients (Hagen et al., 2017; Michel et al., 2002). In keeping with previous research (Lees et al., 2014; Talseth et al., 1999, 2001), our study suggests that suicidal patients want mental health workers to focus less on medical models and approaches in their care for them.

In conclusion, even though there has been an increased focus on suicidality in mental health services, our study illustrates that some mental health workers still need more competence in or should focus more on establishing good connections with suicidal inpatients so they feel a sense of companionship, and providing a more individualized treatment and care. *The National guidelines for prevention of suicide in mental health care* have increased knowledge among staff in psychiatric hospitals, but the guidelines have a disproportionate emphasis on assessment, management of risk factors (such as mental disorders), and suicide risk and lack guidance on several aspects related to good treatment and care. Based on the perspectives of five former hospitalized suicidal persons, mental health workers should first and foremost make efforts to connect with the suicidal inpatients, recognize them as valuable fellow human beings, explore and understand their suicidality, and respond to their particular needs as well as promoting their personal recovery process. Individualized treatment and care of suicidal patients should be given a higher priority among policy makers, researchers, educators, and practitioners, and mental health workers should receive sufficient resources, training and support so they are able to provide the kind of treatment and care suicidal persons seek and need.

The strengths and limitations of the current study need to be acknowledged. This is a small qualitative study based on interviews of five persons. The findings are closely connected to the context in which they were developed but may apply to other similar clinical settings and to other persons in similar situations as the participants; thus, the findings may have a degree of transferability (Malterud, 2001, 2017; Polit & Beck, 2010). However, the assessment of transferability largely depends on the utilitarian value that readers and consumers assign to the research (Polit & Beck, 2010). Further, our findings and descriptions are in keeping with and support previous research (e.g., Berg et al., 2017; Cutcliffe et al., 2006; Sellin et al., 2017; Talseth et al., 1999, 2001). Thus, the findings are generalizable on an analytic level (Polit & Beck, 2010). Future research should further study treatment and care of suicidal inpatients, particularly what good treatment and care means to people with lived experience of suicidality. We need more qualitative research, including field studies in psychiatric hospital wards, where interview data can be complemented with observational data.
